# Habit formation of preventive behaviours during the COVID-19 pandemic: a longitudinal study of physical distancing and hand washing

**DOI:** 10.1186/s12889-022-13977-1

**Published:** 2022-08-20

**Authors:** Chao Zhang, Marieke A. Adriaanse, Renske Potgieter, Lars Tummers, John de Wit, Jan Broersen, Marijn de Bruin, Henk Aarts

**Affiliations:** 1 Department of Psychology, Utrecht University, Utrecht, The Netherlands; 2 Human-Technology Interaction Group at Eindhoven University of Technology, Eindhoven, The Netherlands; 3 Department of Public Health and Primary Care, Leiden University Medical Center, Leiden, The Netherlands; 4 Institute of Psychology, Leiden University, Leiden, The Netherlands; 5 Department of Public Governance and Management, Utrecht University, Utrecht, The Netherlands; 6 Department of Interdisciplinary Social Science, Utrecht University, Utrecht, The Netherlands; 7 Department of Philosophy, Utrecht University, Utrecht, The Netherlands; 8 Radboud University Medical Centre, Njimegen, The Netherlands; 9 National Institute for Public Health and the Environment (RIVM), Bilthoven, The Netherlands

**Keywords:** Habit formation, Habit-intention interaction, Physical distancing, Personal hygiene, Preventive behavior, Longitudinal study, COVID-19

## Abstract

**Background:**

Since the outbreak of the COVID-19 pandemic, physical distancing and hand washing have been used as effective means to reduce virus transmission in the Netherlands. However, these measures pose a societal challenge as they require people to change their customary behaviours in various contexts. The science of habit formation is potentially useful for informing policy-making in public health, but the current literature largely overlooked the role of habit in predicting and explaining these preventive behaviours. Our research aimed to describe habit formation processes of physical distancing and hand washing and to estimate the influences of habit strength and intention on behavioural adherence.

**Methods:**

A longitudinal survey was conducted between July and November 2020 on a representative Dutch sample (n = 800). Respondents reported their intentions, habit strengths, and adherence regarding six context-specific preventive behaviours on a weekly basis. Temporal developments of the measured variables were visualized, quantified, and mapped onto five distinct phases of the pandemic. Regression models were used to test the effects of intention, habit strength, and their interaction on behavioural adherence.

**Results:**

Dutch respondents generally had strong intentions to adhere to all preventive measures and their adherence rates were between 70% and 90%. They also self-reported to experience their behaviours as more automatic over time, and this increasing trend in habit strength was more evident for physical-distancing than for hand washing behaviours. For all six behaviours, both intention and habit strength predicted subsequent adherence (all *ps* < 2e-16). In addition, the predictive power of intention decreased over time and was weaker for respondents with strong habits for physical distancing when visiting supermarkets (*B* = -0.63, *p* <.0001) and having guests at home (*B* = -0.54, *p* <.0001) in the later phases of the study, but not for hand washing.

**Conclusions:**

People’s adaptations to physical-distancing and hand washing measures involve both intentional and habitual processes. For public health management, our findings highlight the importance of using contextual cues to promote habit formation, especially for maintaining physical-distancing practices. For habit theories, our study provides a unique dataset that covers multiple health behaviours in a critical real-world setting.

## Background

The outbreak of the COVID-19 virus has changed the world drastically. In just a few months, several COVID-19 preventive strategies were enforced through the implementation of regulatory decisions and institutional law (e.g., working from home, shutdown of public events and services, mandatory face masks), while other important behavioural measures were recommended to decrease the risk of spreading the virus. In the Netherlands, two most implemented recommendations were *washing hands frequently* and *keeping physical distance from others*. While both hand washing and physical distancing (also called “social distancing) are considered to be effective in slowing down disease transmission [1], they require individuals to autonomously change their behaviours and their effectiveness depends on the public’s adherence to these novel behaviours. Hand washing might be a rather common behaviour in most cultures, but the new preventive measures require people to wash their hands more often, more carefully, and in many new situations (e.g., after coming home from outside, before entering shops). Furthermore, keeping physical distance from other people is not a common practice to most people, so it takes effort and willpower to adapt to and maintain the new behavioural patterns [2].

In this regard, public health management calls for the science of behaviour change and science-based behavioural interventions [3]. According to behaviour change theories (for overviews, see [4, 5]), a major part of human behaviours originate from intentions to engage in them, and requires conscious attention to realize them [6, 7]. Based on classic social cognitive theories (e.g., Theory of Planned Behaviour, [6]; Health Belief Model, [8]), many studies have thus examined and confirmed the roles of behavioural intention, attitude, beliefs (e.g., perceived threats of the virus), social norms, and self-regulatory skills on people’s adherence to preventive behaviours in the COVID-19 context [9–14]. These findings justify interventions that aim at changing intentions, for example, by educating people about the values of physical distancing and hand washing for slowing down the infection rate in the society.

While intentional processes are certainly important for initiating behaviour change, the long-term practice of new behaviours and their maintenance involve other more automatic processes. Specifically, if people have the ability and proper facilities to repeat and maintain the behaviour in the same context, a cognitive link between the context and the behaviour may be strengthened, which facilitates later behaviour selection and execution [15–17]. This habit formation process implies that when behaviour becomes habitual, it can be triggered by and executed in the context at hand without forming intentions and requiring much deliberation or mental effort. From this point of view, habit formation also has implications for behaviour prediction. It is well-known that the strength of one’s habit (or habit strength) predicts future behaviour in addition to behavioural intention [18]. Importantly, previous research shows that strong habits attenuate the predictive value of intention for future behaviour, qualified by a habit-intention interaction effect in predicting behaviour (e.g., [18–24]). The interaction between measures of habit and intention in predicting future behaviour indicates that when strong habits exist, people do not need to deliberate and reason about their behaviour in order to act.

The science of habit formation has also been called upon in the management of preventive behaviours in the COVID-19 context [25], although to a lesser extent than intentional processes. In their prospective paper, Harvey and colleagues discussed intervention strategies that directly focus on promoting habit formation, such as establishing contextual cues, engaging in behavioural repetition, and aiming for automaticity [25]. Some of these strategies have been used in the Netherlands. For example, local and national measures to target physical distancing involved explicit changes in the environment that draw people’s attention to regulate behaviour directly, such as walking stickers in the city centre pedestrian areas and in supermarkets, and specific standing and seating arrangements in shops and restaurants. However, there is no quantitative description of whether Dutch people actually formed habits of physical distancing and hand washing over time (e.g., whether they experienced the behaviours as more automatic over time) and whether the control of the novel behaviours became less intentional but more driven by environmental cues (i.e., effects of intention, habit strength, and their interaction on adherence).

To our best knowledge, only two studies have empirically examined habit formation in the COVID-19 context to some extent. In a cross-sectional study in the UK [26], researchers found self-reported habit strength predicted hand hygiene behaviours. Similarly, a recent prospective survey study found that self-reported habit strength accounted for extra variance in predicting physical distancing behaviours in addition to intention in an Australian and a U.S. sample [27]. These findings suggest that the subjective experience of behavioural automaticity can contribute to predict the degree to which people adhere to preventive behaviours. However, they did not report the interaction between intentions and habits, nor did they track the development of habits over time. In addition, there is no similar research on the Dutch population at a large scale.

To address this knowledge gap, we examined the role of habit formation in the public’s adherence to hand washing and physical distancing recommendations more systematically through a longitudinal study on context-specific behaviours. The longitudinal design allowed us to monitor the changes in habit strength, intention, and adherence to recommendations of physical distancing and hand washing over time, and to examine how habit and intention predict behaviour in response to different phases of the pandemic. Moreover, the inclusion of multiple context-specific behaviours relating to the two preventive measures enabled us to compare habit formation processes across behaviour types and contexts. In theory, behaviour repetition and context stability are two important factors that facilitate habit formation [20]. Influences of these factors can differ greatly not only between hand washing and physical distancing, but also between context-specific behaviours within these two behavioural categories (e.g., hand washing after returning home versus after using toilet; physical distancing in supermarkets versus at home). Obtaining such a quantitative multi-week multi-behaviour picture of habitual and intentional processes underlying the preventive behaviours is potentially useful for policymakers and public health organizations to make more accurate behaviour predictions and to design more effective future interventions [3, 28].

## Methods^1^

### Aims

We report here the results of a 20-week longitudinal study with a large representative Dutch sample (n = 800) on habit formation in their responses to physical-disancing and hand washing measures at the early months of the COVID-19 pandemic. We focused on physical distancing and hand washing, which were further defined as six context-specific behaviours: hand washing *after returning home*, *before eating* and *after using toilet*; physical distancing when *shopping in supermarkets*, when *having guests home* and when *meeting people outside*. We addressed the following research questions. RQ1: How do intention, habit strength, and behavioural adherence change over time for the six context-specific behaviours? We aimed to quantitatively describe the temporal patterns and the differences across the behaviours. RQ2: How do habit strength and intention jointly predict behaviour adherence? We estimated their main and interaction effects in different periods of the pandemic and for the six different behaviours.

### Sample description

One thousand and two hundred respondents were recruited through Panel Inzicht B.V., a major online research panel in the Netherlands. The sample size was determined by our goal of obtaining a large and representative Dutch sample and our budgetary constraints. A subset of the panel received email invitations to participate in the study, if they met the criteria of being a Dutch nationality over 18 years old and living in the Netherlands. A quota sampling was used to obtain a sample that represented the Dutch population on the following demographic variables: age, gender, region of residence and education level. The respondents responded to between 75% and 100% of all weekly surveys, with an average response rate of 97% (*SD* = 0.04). The final sample closely resembled the initial sample in terms of the four variables used in the sampling procedure and other demographic variables (see Table 1 for a detailed description). All respondents explicitly gave their consent to join the longitudinal study.

**Table 1:** Demographics of the initial sample and final sample

Demographic Variable	Initial Sample (*n* = 1200)	Final Sample (*n* = 800)
Age	Between 18 and 90;	Between 18 and 90;
	median = 53, *SD* = 18.09	median = 53, *SD* = 18.09
Gender	600 men, 600 women	398 men, 402 women
Region of Residence^1^	I: 46%; II: 10;	I: 47.8%; II: 9.5;
	III: 21%; IV: 23%	III: 20.4%; IV: 22.4%
Education Level^2^	Low: 16%; Medium: 45%;	Low: 15.5%; Medium: 46.1%;
	High: 39%	High: 38.4%
Employment	Employed: 49.9%;	Employed: 50%;
	Unemployed: 6.6%;	Unemployed: 6.6%;
	Student: 4.6%;	Student: 4.3%;
	Retired: 29.8%;	Retired: 30.5%;
	Other: 9%	Other: 8.6%
Annual Income^3^	Low: 26.9%; Medium: 56.7%;	Low: 24.8%; Medium: 57.3%;
	High: 16.4%	High: 18%
Coronavirus Test^4^	5.1%	3.9%

^1^Region 1 includes the provinces of Noord-Holland, Zuid-Holland and Utrecht; Region 2 includes the provinces of Groningen, Friesland and Drenthe; Region 3 includes the provinces of Overijssel, Gelderland and Flevoland; Region 4 includes the provinces of Zeeland, Noord-Brabant and Limburg

^2^Low education level includes no education, primary school, LBO, VMBO, MBO-1, VBO, MAVO, HAVO or VWO (first three years), VMBO, and (M)ULO; Medium education level includes MBO-2, MBO-3, MBO-4, MBO (before 1998), HAVO or VWO (4th, 5th, or 6th grade), HBS, and MMS; High education level includes HBO (higher applied education) and WO (university undergraduate or above)

^3^Low income level: annual income less than 20k Euro; Medium income level: annual income between 20k and 50k Euro; High income level: annual income more than 50k Euro

^4^Percentage of respondents tested positive before the intake

### Survey design and procedure

**Fig. 1:**
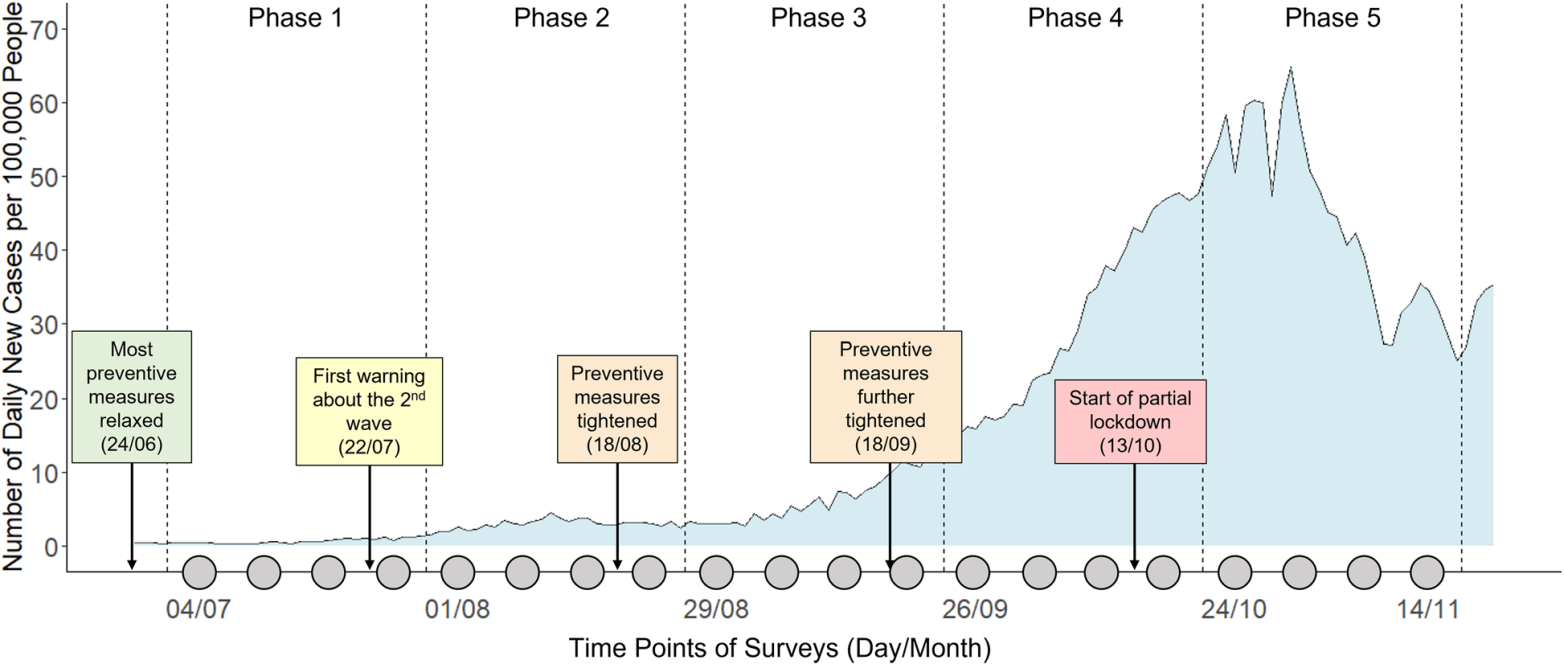
Timeline of the longitudinal survey in the context of the development of COVID19 pandemic in the Netherlands. The 20 waves of the study were aggregated into five phases that roughly followed critical events of policy changes. The curve in the background indicates the number of daily new cases per 100,000 people

The longitudinal survey was conducted between July 4 and November 14, 2020, a period when the initial optimism about the pandemic was followed by the “second wave” of coronavirus in the Netherlands. The 20-week period could be further divided into five distinct phases that corresponded to the development of the pandemic (see Fig. 1 for a visualization of the timeline, different phases, and their contexts). Despite drastic policy changes in this period, the measures of physical distancing and hand washing were always in place. Following previous research on habit formation [20, 21], in each wave, we assessed the respondents’ behavioural intentions, habit strengths, and behaviour adherence for the six context-specific behaviours through self-report. A recent study has shown that self-reports closely match real world behaviours in the COVID-19 context [29].

In each wave, an email invitation was sent to all respondents on Saturday evening at 18:00. Following the invitation, respondents had 24 hours to complete the survey. Initially, a reminder for completing each survey was also sent on Sunday evening at 18:00, but we decided to cancel this reminder after the 10th wave, due to the very high response rate. Respondents used the link in the invitation email to complete the survey implemented on Qualtrics. The approximately 15-minute weekly survey started with questions pertaining to the key variables in this research (i.e., behavioural intention, behaviour adherence, habit strength, and context stability) and then other questions relating to their lives amid the pandemic. After completing each wave of the longitudinal survey, respondents received credits from Panel Inzicht B.V. (equivalent to 70 cents) as compensation. To minimize survey attrition, respondents who managed to complete the whole study received bonus credits (equivalent to 10 euro). After each wave, we checked the validity of the responses and kept track of the respondents who successfully completed the surveys. We excluded those respondents from the study (i.e., they received no further email invitations for subsequent waves) who responded to less than two surveys in each study phase.

### Measurements

For each of the six context-specific behaviours, the same set of questions concerning *behavioural intention*, *behaviour adherence*, *context stability*, and *habit strength* was asked. All questions were either adopted from existing Dutch scales or translated into Dutch by the researchers. Below we use physical distancing when visiting supermarkets as an example to illustrate the specific questions.

Behavioural intention was measured with two items adapted from [20]: “To what extent do you intend to keep 1.5-meter distance when shopping in supermarkets in the coming week?” and “To what extent do you plan to keep 1.5-meter distance when shopping in supermarkets in the coming week?”. Respondents answered the questions using 9-point scales with labels for the end points (e.g., 1 = No, not at all and 9 = Yes, absolutely^2^). The internal reliability of the two-item scale was extremely high for all six behaviours (all Cronbach’s alpha ≥0.97). To assess respondents’ adherence to this behaviour, we asked about context frequency with one question: “How many times did you visit supermarkets in the last week?”. Adherence frequency was measured with one item: “During the supermarket visits you had last week, how many times did you keep 1.5-meter distance from others?”. Respondents indicated their answers to these two questions with numbers. Non-adherence frequency was calculated as context frequency subtracted by adherence frequency, and adherence rate was the ratio between adherence frequency and context frequency. Context stability was measured using one item adapted from [20]: “In situations where you kept 1.5-meter away from others during your supermarket visits last week, to what extent was the context (the moment, the place and the circumstances) different or the same?” (1 = Completely different; 9 = Completely the same).

Finally, based on the literature, we measured habit strength in two different ways. First, we used a traditional measure that indexes habit as a frequency-in-context measure. This measure is based on the idea that higher frequency of behavioural execution and more stable context result in stronger habits. We followed the literature to calculate a habit strength index by multiplying self-reported past behaviour (adherence) frequency and context stability [20, 22, 30, 31]. Second, we included the commonly used Self-Reported Behavioural Automaticity Index (SRBAI; [32]). This measure does not look at frequencies of behaviours, but taps into people’s reflection or metacognition of considering their behaviours as being automatic or effortless. The SRBAI has been used to describe the developments of habits over time [33, 34]. Specifically, a 4-item Dutch version was used in our study (Cronbach’s alpha ≥0.95 for all six behaviours): respondents were asked to rate four statements on 7-point scales (1 = Completely disagree; 7 = Completely agree): “Keeping 1.5-meter away from others when I visit supermarkets is something I do very often.”, “...I do without having to consciously remembering it.”, “...I do without thinking.”, “...I start doing before I realize I am doing it.”. To distinguish the two measures of habit strength, we refer to them as *frequency-based habit index* and *behavioural automaticity* for the remainder of the paper.

### Data analysis

Statistical analyses were performed on data from the 800 respondents who completed the whole longitudinal study. As we were interested in how relationships between variables changed over time, this strategy ensured that for each time period any effects of interest were estimated from the same group of people, making the results more comparable across different time periods. Another general strategy was to aggregate the 20 waves into the five distinctive phases mentioned in Fig. 1 before describing and modelling the data. This decision was based on two considerations. First, while surveying respondents every week could be informative in theory, most variables that were measured did not change that much on a weekly basis. There were also usually no significant changes in the development of the pandemic and the Dutch government’s policies from week to week. Thus, the five phases were more distinctive contextually and they provided a more meaningful backdrop for interpreting the results. Second, aggregating or averaging certain variables over multiple weeks could help to reduce random fluctuations due to measurement issues. This was especially true for frequency-based measures. In a particular week, some respondents might have very limited or no opportunity to perform a specific behaviour (i.e., small or zero context frequency), so adherence and non-adherence frequencies as well as adherence rate were less reliable. For example, if a respondent only visits supermarkets once in a week and adheres to the physical distancing rule, an adherence rate of 1 will be computed but it is not a good measure of how they would behave if more visits are paid. Aggregating frequencies over multiple weeks could attenuate this problem.

For RQ1, we described the means, standard deviations, and intra-class correlations (ICCs) for all the key variables measured, including intention, behavioural automaticity, behaviour frequency, context stability, and adherence rate, and we compare the descriptive statistics across the six context-specific behaviours. The ICCs indicates the proportion of variations in a variable that could be attributed to individual differences. Thus, a higher ICC means relatively large between-person variations compared to within-person changes over time. To describe the aggregated temporal developments of adherence rate, intention, and behavioural automaticity^3^, we visualized the changes of the variables over the five phases of the study and tested linear temporal trends using linear mixed models with time (phase number) as a fixed-effect predictor.

For RQ2, for each phase and each context-specific behavior, we used behavioural intention, habit strength, and their interaction term to predict behavioural adherence in a subsequent period. The outcome variable was the adherence frequency (e.g., frequency of keeping distance in supermarkets) relative to the context frequency (e.g., frequency of visiting supermarkets) in the four weeks of a specific phase. To model this outcome, an intuitive approach would be to model it as adherence rate, i.e., adherence frequency divided by context frequency. However, adherence rate would be a proportion bounded between 0 and 1, so the assumption of ordinary least squares (OLS) regression for continuous variables did not hold. Another problem of computing adherence rate was that the value of the denominator (i.e., context frequency) would be disregarded. For example, an adherence rate of 1 could mean someone adhered to the distancing rule 1 out of 1 time or 10 out of 10 times when visiting supermarkets. Accuracy of model estimation would suffer if these observations were not weighted based on the denominator. For these two reasons, we followed recommendations by statisticians to use logistic regression on a two-vector outcome variable, i.e., adherence frequency versus non-adherence frequency, which could automatically weigh larger samples (the sum of adherence and non-adherence frequencies) more in its estimation [35, 36]. To account for overdispersion, quasi-binomial distributions were assumed.

The predictors were the variables measured prior to the phase used for calculating the outcome variable. For intention, behavioural intention in the first week of each phase was used. The modelling procedure was repeated for the two measures of habit strength. When frequency-based habit index was used, for each phase, the product of total behaviour frequency and average context stability in the weeks prior to the start of the phase was used. When behavioural automaticity was used, its measurement in the first week of each phase was used. Before computing the interaction term, both behavioural intention and habit strength were grand-mean centred for each phase and standardized to facilitate the interpretation of the results.

We also checked whether intention correlated with the two different measures of habit strength and whether strong correlations created multicollinearity issues for the regression models [37]. For frequency-based habit index, multicollinearity was not an issue at all, given the moderate correlations between intention and this habit strength measure (Pearson’s *r* between 0.21 and 0.40). In contrast, behavioural automaticity correlated strongly with intention for all six behaviours in all five phases (Pearson’s *r* between 0.73 and 0.80). The Variance Inflation Factors (VIFs) were in many cases approaching but still under the threshold for multicollinearity (i.e., 5), with the exception that for physical distancing when visiting supermarkets in the last phase (VIF = 6.99 and 5.82 for behavioural automaticity and the interaction term). Still, the high correlations between intention and behavioural automaticity were not ideal for testing their interaction effect on behavioural adherence (see [38, 39]). Therefore, while the modelling results using the two different habit strength measures were similar and we report both in the Results section, the results based on the frequency-based measure were likely to be more reliable.

For all statistical tests, we set the alpha level at 0.00083 given that we tested each effect for six behaviours, five phases and two different measures of habit strength (0.05 divided by 60). To give an indication of statistical power, our sample size of 800 was sufficient to detect a bivariate correlation of *r* = 0.163 with 90% power. All analyses were performed in the R statistical computing environment, version 4.1.1 [40].

## Results

### Descriptives and temporal developments of behaviour adherence, intention, and habit strength

Tables 2 and 3 show the means, standard deviations, and ICCs of the measured variables. Our Dutch sample reported to have strong intention to adhere to all the six context-specific behaviours (all means above 7.39 on 9-point scales). In addition, the very high ICCs (all above 0.87) indicated that the variations in intentions were predominately accounted for by individual differences among the survey respondents rather than temporal variations within individual persons. Based on scores for the behavioural automaticity index, survey respondents self-reported to have moderate to strong habits for the six behaviours (all means above 5 on 7-point scales). As with intention, ICCs indicated much larger differences in behavioural automaticity between respondents than within respondents over time (all ICCs ≥0.87). For both intention and behavioural automaticity, the same order from larger to smaller means emerged for the six behaviours – hand washing after using toilet, physical distancing when visiting supermarkets, hand washing after returning home, hand washing before eating, physical distancing when meeting people outside, and physical distancing when having guests home.

**Table 2:** Grand means, standard deviations (*SD*), and intra-class correlations (ICC) of the key measures pertaining to physical distancing behaviours. The descriptives were calculated after aggregating the data into five phases

	Physical distancing					
	*Visiting supermarket*		*Having guests home*		*Meeting people outside*	
	Mean (*SD*)	ICC	Mean (*SD*)	ICC	Mean (*SD*)	ICC
Behavioral intention	7.89 (1.59)	0.87	7.39 (2.00)	0.89	7.58 (1.85)	0.88
Behavioral automaticity	5.51 (1.47)	0.87	5.02 (1.69)	0.88	5.20 (1.61)	0.88
Context frequency	2.56 (2.18)	0.83	1.24 (1.67)	0.51	1.27 (1.55)	0.63
Adherence frequency	2.26 (2.35)	0.86	0.95 (1.56)	0.66	1.02 (1.42)	0.56
Adherence rate	0.88 (0.25)	0.70	0.71 (0.37)	0.75	0.76 (0.33)	0.73
context stability	7.15 (1.87)	0.73	7.34 (1.89)	0.66	6.65 (2.12)	0.60

**Table 3:** Grand means, standard deviations (*SD*), and intra-class correlations (ICC) of the key measures pertaining to hand washing behaviours. The descriptives were calculated after aggregating the data into five phases

	Hand washing					
	*After returning home*		*Before eating*		*After using toilet*	
	Mean (*SD*)	ICC	Mean (*SD*)	ICC	Mean (*SD*)	ICC
Behavioral intention	7.74 (1.86)	0.92	7.60 (1.90)	0.92	8.34 (1.34)	0.93
Behavioral automaticity	5.34 (1.71)	0.92	5.32 (1.75)	0.93	6.19 (1.27)	0.91
Context frequency	10.21 (8.18)	0.82	20.81 (8.09)	0.72	31.93 (17.18)	0.88
Adherence frequency	8.46 (8.01)	0.80	16.25 (9.75)	0.70	29.36 (17.20)	0.86
Adherence rate	0.82 (0.27)	0.86	0.76 (0.32)	0.87	0.91 (0.21)	0.88
context stability	7.21 (1.79)	0.78	7.48 (1.60)	0.78	7.57 (1.63)	0.79

In terms of self-reported behaviour, adherence frequencies of the six behaviours differed substantially, but the differences were mainly attributed to the differences in people’s opportunities to perform physical distancing and hand washing in specific contexts (i.e., context frequency). For example, respondents had more opportunities to practice the hand washing behaviours than the physical distancing behaviours. Within the category of physical distancing, they practiced the behaviour more often when visiting supermarkets than when meeting people outside or having guests at home. When taking the differences in context frequency into account, adherence rates of the behaviours were close to each other and respondents on average adhered to all of the recommendations in the majority of the time (all means above 70%). Again, respondents differed a lot from each other in their adherence rates, but their own adherence rates were relatively stable over the 20 weeks (all ICCs ≥ 0.7). Finally, respondents reported to practice all the behaviours in relatively stable contexts (all means ≥ 6.65). Still, behaviours practiced at home were associated with higher context stabilities than behaviours practiced outside one’s home.

**Fig. 2:**
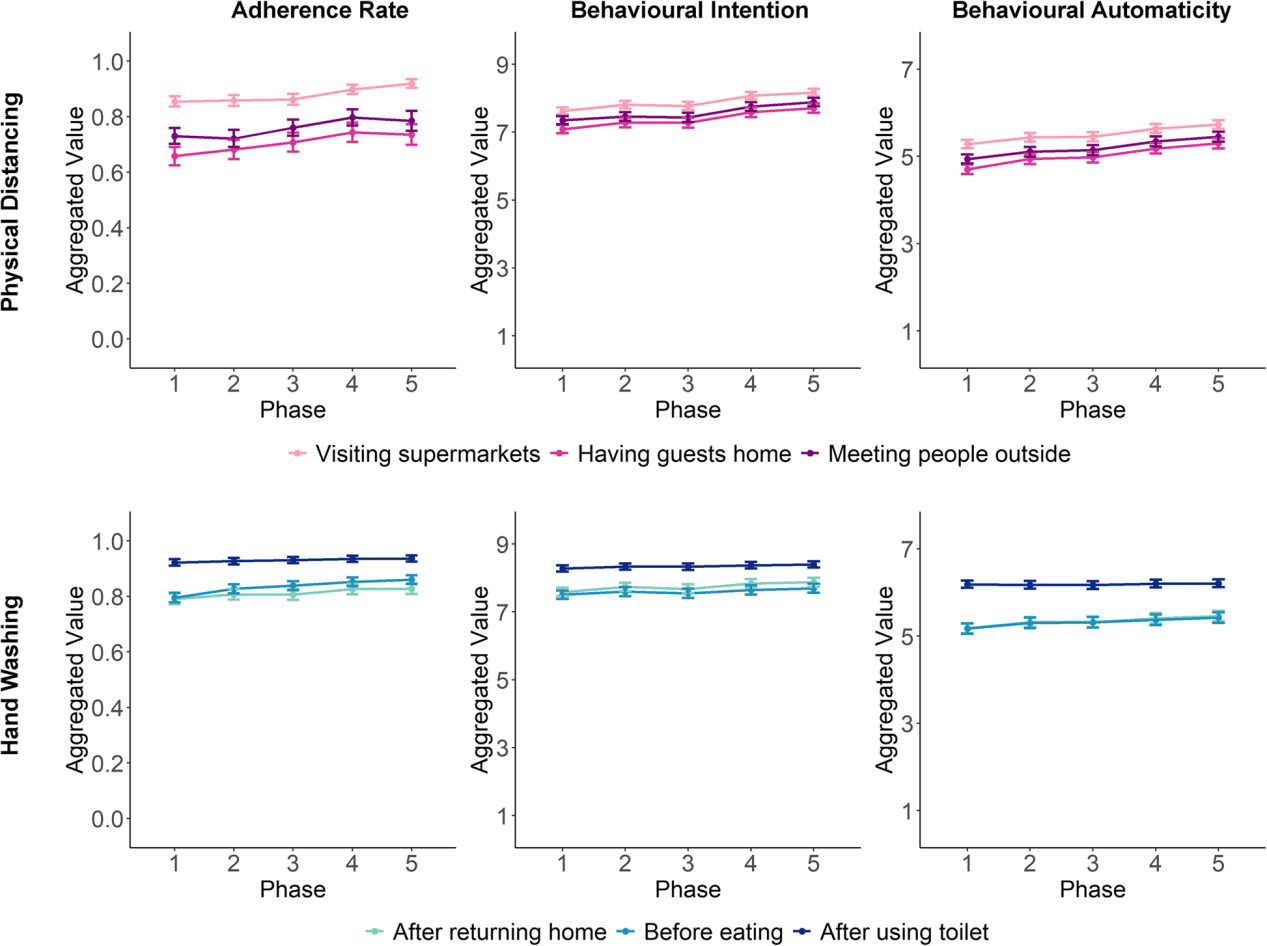
Aggregated temporal developments of adherence rate, behavioural intention, and behavioural automaticity over the five phases of the study

While individual differences accounted for most of the variance in adherence rate, intention, and behavioural automaticity, there were small temporal developments in these variables and clear differences among the six behaviours (see Fig. 2). Adherence rates for the three physical distancing behaviours increased over the five phases, especially in the last two phases (visiting supermarkets: *B* = 0.017, 95% CI = [0.014, 0.020], *p* < 2e-16; having guests home: *B* = 0.023, 95% CI = [0.017, 0.028], *p* <.0001; meeting people outside: *B* = 0.019, 95% CI = [0.014, 0.025], *p* <.0001). Similar but smaller increases were also evident for hand washing behaviours (after returning home: *B* = 0.009, 95% CI = [0.007, 0.011], *p* < 2e-16; before eating: *B* = 0.014, 95% CI = [0.012, 0.017], *p* < 2e-16; after using toilet: *B* = 0.004, 95% CI = [0.003, 0.006], *p* <.0001).

Accompanying the increases in adherence rates, respondents’ intentions to comply with the six behaviours also became slightly stronger over time for both physical distancing (visiting supermarkets: *B* = 0.145, 95% CI = [0.123, 0.147], *p* < 2e-16; having guests home: *B* = 0.154, 95% CI = [0.141, 0.168], *p* < 2e-16; meeting people outside: *B* = 0.136, 95% CI = [0.123, 0.149], *p* < 2e-16) and hand washing (after returning home: *B* = 0.067, 95% CI = [0.056, 0.079], *p* < 2e-16; before eating: *B* = 0.042, 95% CI = [0.031, 0.054], *p* <.0001; after using toilet: *B* = 0.028, 95% CI = [0.020, 0.036], *p* <.0001).

Finally, behavioural automaticity showed a relatively steeper increase for physical distancing behaviours (visiting supermarkets: *B* = 0.109, 95% CI = [0.098, 0.120], *p* < 2e-16; having guests home: B = 0.143, 95% CI = [0.131, 0.155], *p* < 2e-16; meeting people outside: *B* = 0.126, 95% CI = [0.114, 0.138], *p* < 2e-16) than for hand washing behaviours after returning home (*B* = 0.064, 95% CI = [0.053, 0.074], *p* < 2e-16) and before eating (*B* = 0.058, 95% CI = [0.048, 0.068], *p* < 2e-16). Washing hands after using toilet was the only behaviour that did not show an increase in behavioural automaticity over time (*B* = 0.007, 95% CI = [-0.001, 0.015], *p* =.105).

### Predicting future behaviour adherence using intention, habit strength and their interaction

#### Modelling results with frequency-based habit index

**Fig. 3:**
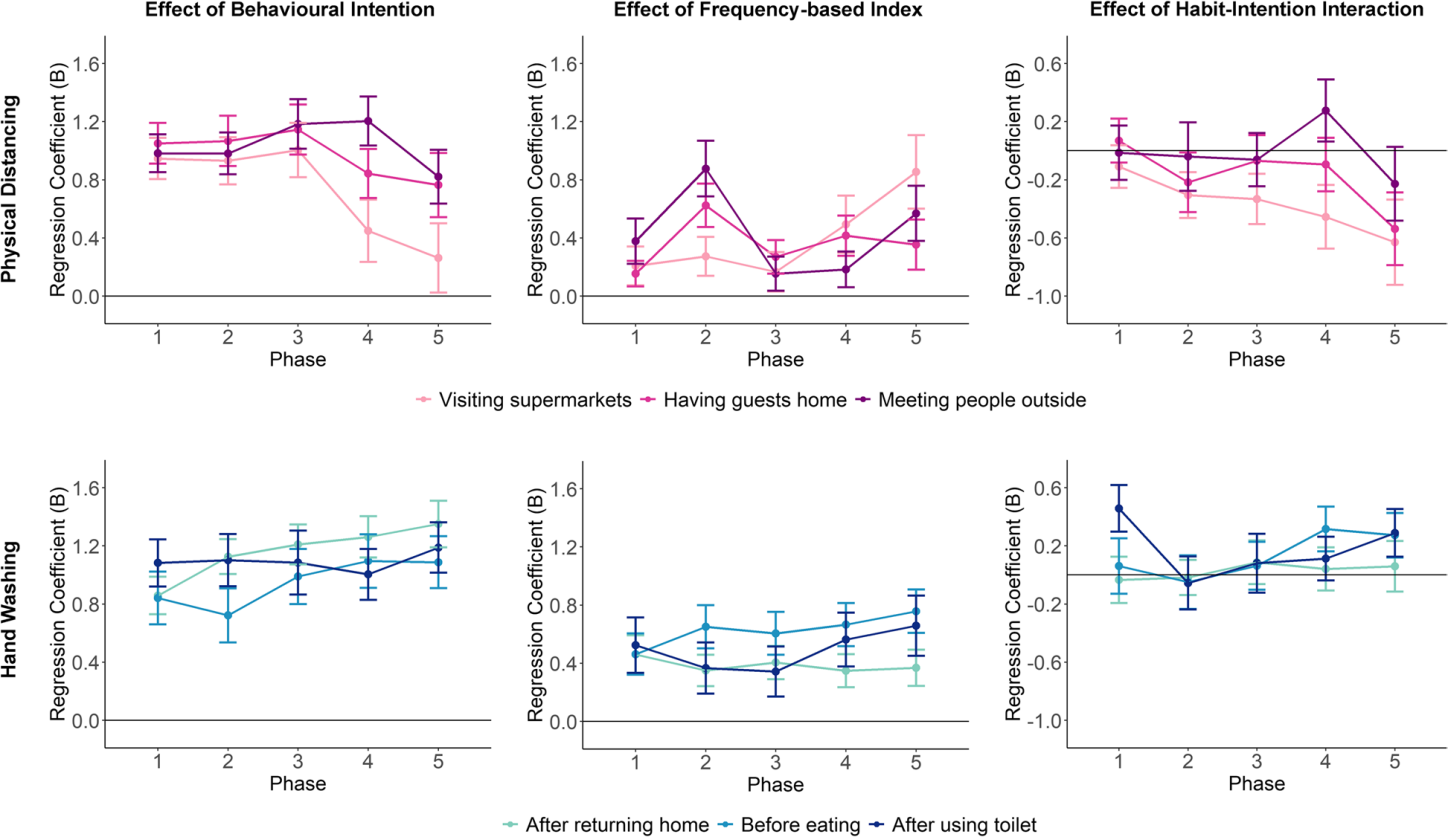
Effects of behavioural intention, frequency-based habit index, and their interaction on subsequent behavioural adherence estimated for each context-specific behaviour at each phase of the study (Regression coefficients on the y-axis represents effect sizes. Error bars represent 95% confidence intervals of the estimates)

Figure 3 shows the effects of intention, frequency-based habit index and their intention term on behavioural adherence for the six context-specific behaviours and over the five study phases. For most behaviours and phases, the estimated effects revealed that respondents with a stronger intention to follow the preventive measures also adhered to those measures to a greater extent. These were relatively large and highly robust correlations, with 95% confidence intervals very far from the reference line of zero (i.e., most *p*-values smaller than 2e-16). Frequency-based habit index also predicted subsequent adherence: respondents who practiced those behaviours more frequently in more stable contexts were more likely to adhere to the recommended behaviours in the future. The effect sizes of frequency-based habit index were smaller than those of behavioural intention.

There was a clear difference in the temporal developments of the effects of intention between physical-distancing and hand washing behaviours. For physical-distancing behaviours, the influences of behavioural intention on subsequent adherence decreased drastically in the last three phases, when the second wave of the pandemic hit the Netherlands and physical-distancing rules were enforced more strictly. This was especially true for physical distancing in supermarkets, for which behavioural intention no longer had significant influence on adherence in the last phase (*B* = 0.26, 95% CI = [0.02, 0.50], *p* =.031). In contrast, the effects of behavioural intention on subsequent adherence showed a small increasing trend for all hand washing behaviours. There was no linear trend in the temporal developments of the effects of frequency-based habit index on adherence, but for physical distancing behaviours, these effects were relatively larger in Phase 2 and Phase 5.

**Fig. 4:**
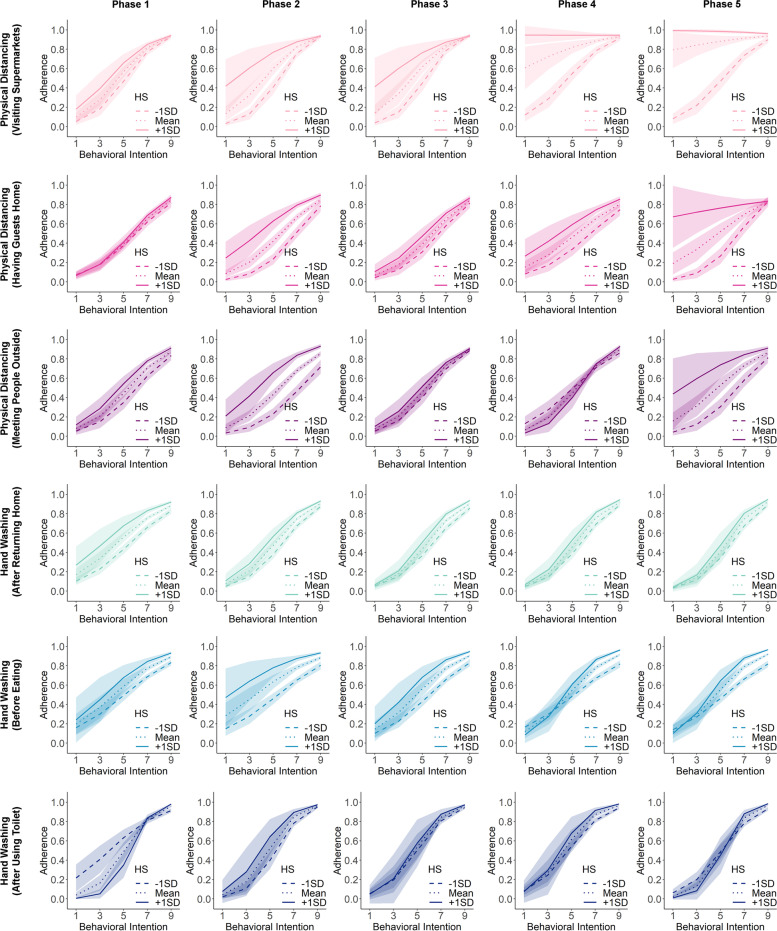
Visualization of the effect of behavioural intention on subsequent behavioural adherence for respondents with different levels of habit strength (HS) measured by frequency-based habit index for each context-specific behaviour at each phase of the study

Modelling analyses also revealed negative habit-intention interaction effects for physical distancing behaviours in the final phases. The effect that stood out specifically was the negative interaction for physical distancing when visiting supermarkets, which became stronger (i.e., more negative) throughout the study phases (Phase 1: *B* = -0.11, 95% CI = [-0.26, 0.04], *p* =.146; Phase 2: *B* = -0.31, 95% CI = [-0.46, -0.15], *p* =.0002; Phase 3: *B* = -0.33, 95% CI = [-0.51, -0.16], *p* =.0002; Phase 4: *B* = -0.45, 95% CI = [-0.67, -0.23], *p* <.0001; Phase 5: *B* = -0.63, 95% CI = [-0.92, -0.34], *p* <.0001). The negative interaction effect was also significant in the last phase for physical distancing when having guests at home (*B* = -0.54, 95% CI = [-0.79, -0.29], *p* <.0001). These interaction effects indicated that for respondents who engaged in these physical-distancing behaviours more frequently in the past and in more stable contexts, their behavioural intentions played a lesser role in predicting their behaviours in the subsequent time period (see Fig. 4 for the visualization of the interaction effects). In contrast, for hand washing behaviours, there was no evidence that for respondents with higher frequency-based habit strengths, their behaviors were less predicted by their behavioural intentions. Instead, there were positive albeit very small interaction effects in the last phases of the study for hand washing after using toilet and before eating.

#### Modelling results with behavioural automaticity

**Fig. 5:**
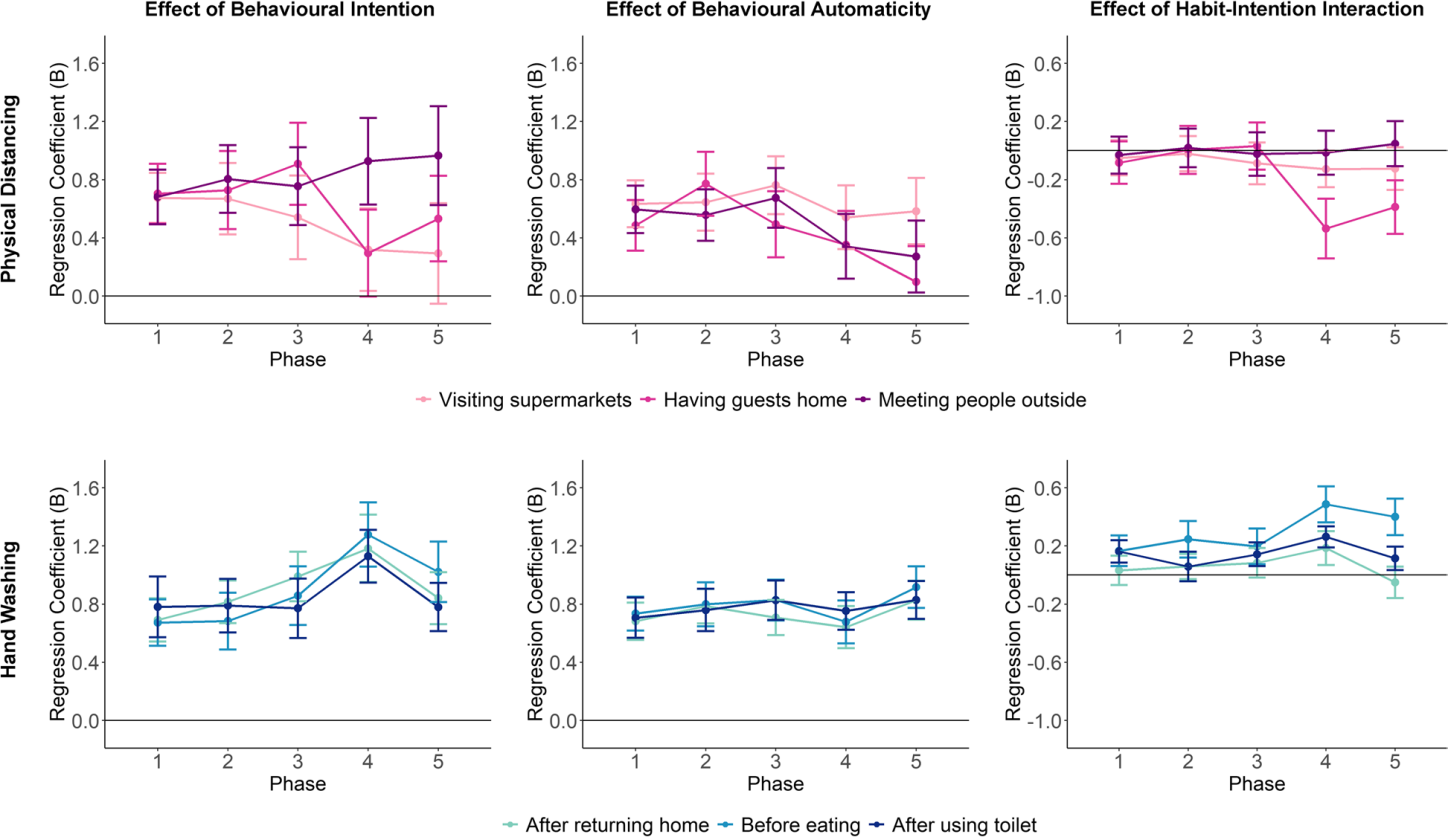
Effects of behavioural intention, behavioural automaticity, and their interaction on subsequent behavioural adherence estimated for each context-specific behaviour at each phase of the study (Regression coefficients on the y-axis represents effect sizes. Error bars represent 95% confidence intervals of the estimates)

The modelling results when behavioural automaticity was used as a predictor were similar to those in the previous section in general. As shown in Fig. 5, both intention and behavioural automaticity were positively associated with future behavioural adherence for all behaviours and all phases (most *p*-values smaller than 2e-16) and their effect sizes were close to each other. The effects of behavioural automaticity implied that for respondents who experienced their behaviours as more automatic and effortless, the more they adhered to the recommendations in the future. There were fewer clear patterns in the temporal variations of these effect estimates.

**Fig. 6:**
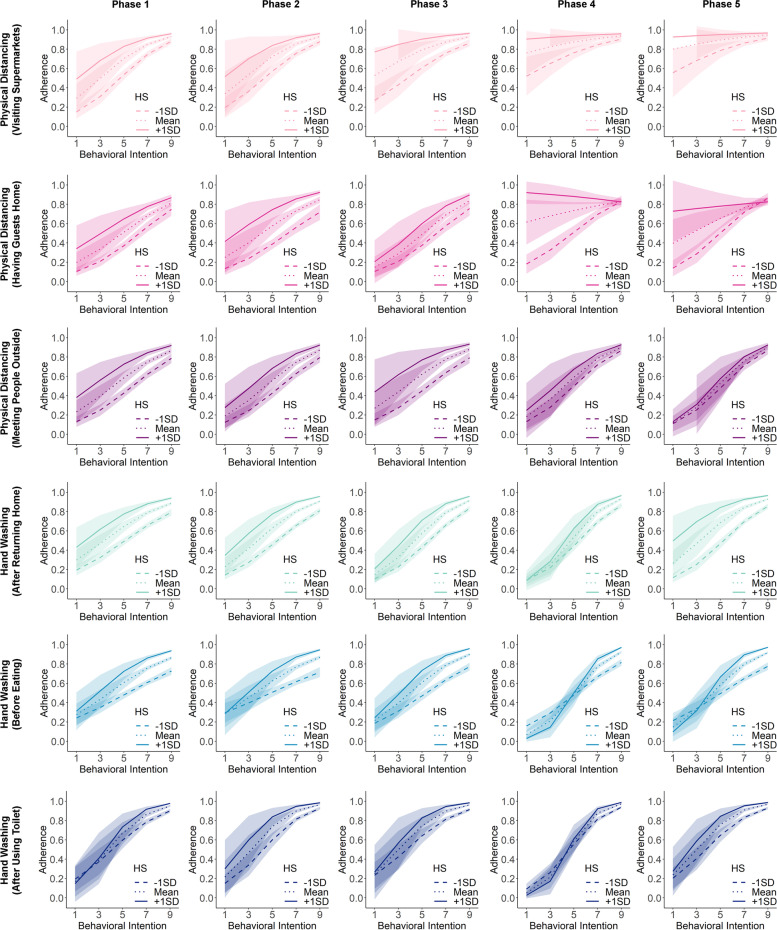
Visualization of the effect of behavioural intention on subsequent behavioural adherence for respondents with different levels of habit strength (HS) measured by SRBAI for each context-specific behaviour at each phase of the study

In terms of the interaction effect between intention and behavioural automaticity, the results only showed a negative interaction effect for physical distancing when having guests at home in the last two phases of the study (see Fig. 6). For respondents who reported to experience stronger behavioural automaticity, their intentions predicted their adherence to a lesser degree (Phase 4: *B* = -0.54, 95% CI = [-0.74, -0.33], *p* <.0001; Phase 5: *B* = -0.39, 95% CI = [-0.57, -0.20], *p* <.0001). For physical distancing when visiting supermarkets, the estimated interaction effects were negative, but not statistically significant at the alpha level we set (Phase 4: *B* = -0.13, 95% CI = [-0.25, -0.002], *p* =.048; Phase 5: *B* = -0.12, 95% CI = [-0.27, 0.02], *p* =.099). For hand washing behaviours, the interaction effects were either close to zero or even slightly positive in the later phases.

## Discussion

### Summary of findings

The present paper reports the findings of a longitudinal study at the early stage of the COVID-19 pandemic in the Netherlands (from June till November 2020, 20 waves of data) on habit formation for physical distancing and hand washing in various contexts, amongst a representative Dutch sample (n = 800). Overall, adding to recent studies [26, 27], our study provides further empirical evidence that habit formation plays an important role in people’s behavioural responses to preventive measures in the context of the COVID-19 pandemic, in addition to the more frequently studied intentional processes such as beliefs and perceived social norms [9–14]. Habit strength, as measured both by the frequency-based habit index and behavioural automaticity measure, predicted subsequent behavioural adherence on top of and beyond behavioural intention. The convergence of both habit measures in predicting behaviour is consistent among the six context-specific behaviours and across several distinct stages of the development of the pandemic in the Netherlands.

Our results further revealed interesting behavioural and psychological differences in the ways Dutch people responded to the two different preventive measures. Compared with the respondents’ stable behavioural patterns regarding the hand washing measures, their adherence to physical-distancing measures in various contexts increased more clearly over time, accompanied by their metacognitions that the initially novel behaviours became more automatic over time. In terms of how habit and intention predict future behaviour, the physical distancing behaviours but not hand washing behaviours were associated with an attenuation of the influence of intention over time among people who formed stronger habits as measured by the frequency-based habit index or the self-reported behavioural automaticity index. The latter interaction effect suggests that for people with stronger physical distancing habits, their future adherence to the measures were less correlated with their intentions to follow those measures. In contrast, while respondents practiced hand washing frequently and experienced the behaviours to be automatic, their behaviours remained to be highly predicted by their intentions. These contrasting results corroborate a recent study that identified distinct predictors for physical distancing versus personal hygiene behaviours among university students in the Netherlands and other European countries [41].

### Implications for public health management regarding the preventive measures

Ours findings support the recent proposal to harness the power of habit formation to increase the public’s adherence to COVID-19 preventive measures [25]. Except for a few behaviours that are enforced by law in the Netherlands and other countries (e.g., wearing a face mask indoor, closing of shops and restaurants), many recommended behaviours to prevent the spread of the coronavirus largely rely on people’s own willingness and abilities to adhere to the recommendations. Thus, changing people’s pre-pandemic behaviours in the latter category and maintaining the recommended behavioural patterns is more difficult and requires the integration of a variety of behaviour change techniques [5, 42]. Successful strategies are likely those that incorporate not only techniques that rely on deliberative and intentional process (e.g., correcting false beliefs about the pandemic, reinforcing behaviours with rewards) but also more environment-based techniques, such as establishing stable contextual cues [20] and implementing context-based plans [43–45]. We recognize that many environment-based techniques have been in place since the start of the pandemic in many countries. For example, in the Netherlands, walking stickers are used in supermarkets to remind people of physical distancing in the situ and consequently, the behaviour of keeping distance may be automatically triggered by such cues. Our data suggest that even the entirely new and unnatural behaviours of physical distancing before the pandemic became automatic to some extent after eight months of practice.

Given the ongoing vaccination campaigns that hold the promise of containing the pandemic in the foreseeable future, perhaps a more timely question is to ask how our findings provide insights into the management of physical distancing and personal hygiene in the near future. For example, an intriguing question is whether people will or should maintain some of the preventive behaviours. For hand washing behaviours, the consistent strong correlation between intention and behaviour may suggest that hand washing routines require sustained intention to perform and execute the behaviour. In case the pandemic will wax and wane, continuous recommendation and education may be needed to maintain strong intention to wash one’s hands in critical settings. As for physical distancing, the attenuated intention-behaviour link suggests that people might be directly guided by the environment to keep distance without paying much attention to it. Accordingly, changing the environment or removing the physical cues may lead to a breakdown of newly formed habits. If we want people to readily and consistently keep physical distance (or to engage in similar preventive behaviours), policies to encourage such new habits should resort to interventions that take advantage of the current social context and environmental cues to direct behaviour at hand.

### Implications for research on habit formation

The interplay between habitual and intentional control of behaviour, such as examined by the habit-intention interaction effect in basic and applied behavioural research, has been considered a central hypothesis predicted by contemporary habit theories [17, 18, 24, 46, 47]. In this regard, our study provides unique data for the habit-intention interaction effect for six different context-specific behaviours in one large study. Interestingly, even though habits as measured by behavioural automaticity were strong for both preventive behaviours, the widely theorized negative habit-intention interaction effect was only evident in some physical-distancing behaviours. This concurs with the recent analysis and debate about whether and how habit weakens the relationship between intention and behaviour [48, 49]. While the interaction effect (or the lack thereof) is based on correlational data, this surprising contrast may suggest different mechanisms of self-regulating the two different behaviours. Below we discuss two speculations.

One difference between physical distancing and hand washing is the level of conscious attention required to control behaviour [50]. The stronger interaction effect for physical distancing behaviours may represent an instance where the environment strongly controls behaviour. That is, once instigated, the actual implementation of behaviour requires little effort or thinking in the environment at hand, for example, the use of warning tapes on the floor or the presence of others in one’s personal space. In contrast, to closely follow hand washing recommendations, one needs to execute a series of actions and monitor their effects, such as opening the tap, using soap, specific hand movements, and keeping an eye on the duration. The execution of these actions may require more attention and remain largely intentional, even when the act of washing hands has been frequently instigated by the same environment.

Another intriguing possibility why physical distancing and hand washing differ in their regulations concerns the question of whether the preventive measures are regulated in private or social contexts. While washing hands properly was strongly recommended, the behaviour is usually performed in private contexts and without external enforcement. Physical distancing, on the contrary, represents a new social norm that was much more strictly enforced than the “self-imposed norm” of washing hands. Because of its social nature, physical distancing might therefore obey different dynamics when it comes to intentions and action repetition [51]. People react and mimic each other and may readily share similar views on the importance of behaviour [52, 53]. This stronger social influence on physical distancing may have rendered it more sensitive to the attenuation of intention-behaviour link when habits are strong.

### Limitations

When interpreting our findings and considering their implications, one should be aware of the limited time period that the behaviours were studied. While our 20-week longitudinal study is relatively extensive, it only captured a specific period of the COVID-19 pandemic in the Netherlands. For example, we could not start our study earlier than the end of June, when the pandemic had hit the Netherlands for three months and, apart from disrupting society at large, preventive measures were already in place. We therefore were unable to capture the initial stage of people’s responses to the measures, a period that might have been critical in adhering to the new rules and forming habits. Moreover, as our study completed in November 2020, no information is available regarding how our respondents’ behaviours might have changed afterwards. Based on the 2021 monitor survey by the National Institute for Public Health and the Environment, we do know by now that Dutch people fail to maintain physical distancing behaviours after May 2021 when measures were temporarily relaxed and environmental cues (e.g., markers in supermarkets) were removed^4^.

Another limitation is that all variables in our study were measured by self-reports that depend on people’s ability to introspect on past experiences. Although we do not have direct evidence, it is known that such subjective reports are biased and inaccurate [54–57]. We aimed to reduce inaccuracies and measured adherence in a well-defined and detailed way by asking respondents to recall the frequencies of encountering specific contexts (e.g., visiting supermarkets) and the frequencies of executing the associated behaviours (e.g., keeping distance from others) in the previous week. While our approach may lead to more precise and less socially biased measures than ratings scales [26, 27], it relies on people’s ability to accurately recall the events in their lives over a period of one week. In general, although self-report is unavoidable for measuring psychological variables (e.g., intention, metacognition of automaticity), actual behaviours can potentially be measured objectively. For example, several sensor systems have been implemented to monitor violations of physical distancing rules (e.g., [58, 59]). In our own recent study, we also tried to use pressure-sensors attached to soap bottles to measure participants’ hand washing behaviours at home. Future studies should explore the advantages and disadvantages of measuring preventive behaviours using sensor technologies and linking them to self-reported measures.

Furthermore, our self-reported measure of context stability may suffer from a more specific issue. Because the question about context stability refers to individual weeks, week-level stability may fluctuate a lot and misrepresent long-term stability, which is more relevant for habit formation. This issue is especially salient for physical-distancing in supermarkets and when a weekly frequency of visiting is low. For example, when some visits two supermarkets in a week, one they usually go to and one for the first time, the weekly self-reported stability would be low but in fact their long-term shopping behaviour is relatively stable. This limitation adds noises to the measure of frequency-based habit index.

Finally, it should be noted that all the effects estimated in our models, including the habit-intention interaction effects, were based on observational data generated from the longitudinal study. Even though we ensured that all predictors (i.e., intention, habit strength) were measured prior to the outcome variable (i.e., adherence rate) in time, strong conclusions about their causal relationships should be avoided. The causal relationships between intention, habit strength and actual behaviour can be complex and bidirectional. For example, not only do strong habits make recurring behaviours more likely by automatically triggering them in the corresponding contexts, but repeating behaviours also lead to stronger habits. The real-life nature of our study has its limits in allowing for a causal test, as this would require for instance experimental manipulations of habit formation that build on frequency and stability of behaviour. In order to formally test hypotheses following habit theories under such limited test circumstances, empirical data could be compared with simulated behavioural patterns generated from computational models of habit formation (e.g., [60–63]; for a review, see [64]).

## Conclusions

The unpreceded pandemic of COVID-19 has made behaviour change a salient problem for public health management. Given the rarity of such events, we have very little empirical knowledge about what factors determine behavioural responses to preventive measures in the context of a pandemic and what works best as management strategies. As such, the ongoing challenge offers a rare opportunity to test behavioural change theories in a highly critical and relevant setting and at a large scale. Our 20-week longitudinal study with a large representative sample provides initial evidence that behavioural adaptation to new physical distancing and hand washing recommendations is matter of both a habit formation process and an intentional effort. What the present study teaches us the most, we believe, is that humans can form habits for completely new behaviours, and that it is worth to consider whether and how such new habit formation can serve society at large when it comes to public health.

## Data Availability

Survey data, data analysis scripts, and documents containing complete lists of survey questions are available at Open Science Framework (https://osf.io/4utk8/).

## Footnotes

1 The study design and data analysis plan were preregistered at open science framework: https://osf.io/kje5v.

2 The English translations of the questions and the labels may sound odd, but the original Dutch versions were intuitive for Dutch speakers. Interested readers can refer to the original questionnaires shared at Open Science Framework: https://osf.io/4utk8/.

3 It is uninformative to show the temporal development of frequency-based habit index. This index is bounded by definition to increase almost linearly over time because it reflected the accumulation of behaviour frequency over time, multiplied by context stability.

4 You can find an online report from the National Institute for Public Health and the Environment here: https://www.rivm.nl/gedragsonderzoek/trendonderzoek
